# Prevalence of Gastroparesis and the Impact of Metformin in Diabetic Patients: A Cross-Sectional Study in Riyadh, Saudi Arabia

**DOI:** 10.1155/grp/3713569

**Published:** 2024-12-04

**Authors:** Jehad A. Aldali, Mushabbab K. Asseri, Haneen A. Almufarrij, Aroob M. Alromih, Albandari M. Alajlan, Khawlah A. Alrashed, Atheer I. ALghadeer, Bushra I. Almutawa, Abdulrahman Alshalani

**Affiliations:** ^1^Department of Pathology, College of Medicine, Imam Mohammad Ibn Saud Islamic University (IMSIU), Riyadh 13317, Saudi Arabia; ^2^Department of Endocrine & Diabetes, Prince Sultan Medical Military City, Riyadh 11159, Saudi Arabia; ^3^Medical School, College of Medicine, King Saud Bin Abdul Aziz University for Health Sciences (KSAU-HS), Riyadh 11481, Saudi Arabia; ^4^Medical School, College of Medicine, Imam Mohammad Ibn Saud Islamic University (IMSIU), Riyadh 13317, Saudi Arabia; ^5^Chair of Medical and Molecular Genetics Research, Department of Clinical Laboratory Sciences, College of Applied Medical Sciences, King Saud University, Riyadh, Saudi Arabia

**Keywords:** diabetes mellitus type 1, diabetes mellitus type 2, gastroparesis, Gastroparesis Cardinal Symptom Index (GCSI), metformin, score

## Abstract

**Background:** The prevalence of gastroparesis in individuals with diabetes mellitus varies significantly across different studies. This study is aimed at estimating the prevalence of gastroparesis among diabetic patients in Riyadh, Saudi Arabia, and evaluating the association between metformin use and clinical manifestations of gastroparesis.

**Methods:** This cross-sectional study employed an online survey distributed via Google Forms, targeting patients at a diabetes clinic. The survey comprised three sections, including the Gastroparesis Cardinal Symptom Index (GCSI). Eligible participants were those diagnosed with either type 1 or type 2 diabetes mellitus and aged 18 or older.

**Results:** The study included 385 participants, with the majority diagnosed with type 2 diabetes (55.6%) for over 10 years (59.5%). A significant proportion had poorly controlled blood glucose levels (56.6%) and were taking metformin (50.9%). Among gastrointestinal (GI) symptoms, “stomach fullness” was reported most frequently (53.2%), whereas “vomiting” was reported least often (17.9%). GCSI scores did not differ significantly between type 1 and type 2 diabetes patients (*p* = 0.88). However, patients with diabetes durations of less than 3 years, those with durations of 5–7 years controlled blood glucose levels, and those on metformin exhibited higher GCSI scores (*p* = 0.20, *p* = 0.02, and *p* = 0.10, respectively).

**Conclusion:** This study identified some commonalities as well as differences in the prevalence and symptomatology of gastroparesis among diabetic patients. We observed no significant variation in GCSI scores between type 1 and type 2 diabetes. Nevertheless, higher GCSI scores were associated with shorter diabetes durations, controlled blood glucose levels, and metformin use. However, due to the small sample size and reliance on self-reported data, one should interpret the study's findings with caution.

## 1. Introduction

Diabetes mellitus (DM), commonly referred to as diabetes, is a chronic metabolic disorder characterized by persistent hyperglycemia resulting from defects in insulin secretion, insulin action, or both [1]. A study by Wang, Fisher, and Parkman, which investigated trends in gastroparesis-related hospitalizations from 1995 to 2004, found that 53% of the increasing risk of diabetes-related hospitalizations was attributable to gastroparesis [2]. Gastroparesis, also known as delayed gastric emptying (GE), is a condition in which the stomach's ability to move food into the small intestine is impaired, despite the absence of mechanical obstruction [1]. This condition, a form of autonomic neuropathy, predominantly affects individuals with diabetes who have experienced the disease for over a decade and who have significant microvascular complications [3]. Symptoms commonly associated with gastroparesis include early satiety, nausea, bloating, abdominal discomfort, and vomiting. Notably, these symptoms may persist and stabilize over a period of 12 to 25 years, even with controlled blood glucose levels [4]. Elevated blood glucose levels can induce nerve damage, thereby impairing the function of gastric muscles and contributing to the onset of gastroparesis [5].

The pathophysiology of diabetic gastroparesis involves dysfunction of the enteric and autonomic nervous systems. Chronic hyperglycemia induces neuronal damage, resulting in aberrant myenteric neurotransmission, impaired inhibitory (nitric oxide) neuronal function, and dysfunction of smooth muscle and interstitial cells of Cajal (ICC) [6]. The loss of ICC, which serves as an electrical pacemaker and facilitates neuromuscular activity in the stomach, is particularly implicated in the onset of diabetic gastroparesis [7, 8].

Epidemiological data suggest that approximately one-third of gastroparesis cases are attributable to diabetes. The incidence of gastroparesis in the United States is estimated to be 5.2% in type 1 diabetes mellitus (T1DM) patients over a decade and 1% in type 2 diabetes mellitus (T2DM) patients [9, 10]. On the other hand, the prevalence of gastroparesis in T2DM was 6% in Saudi Arabia [11]. Notably, the use of incretin mimetics in T2DM management may exacerbate the risk of gastroparesis. The age-adjusted incidence rates of gastroparesis are reported as 9.8/100,000 person-years for females and 2.4/100,000 person-years for males [12]. GI symptoms are prevalent in 5%–12% of individuals with diabetes, with upper GI symptoms present in 11%–18% of diabetic patients, predominantly correlating with delayed GE [13]. The chronicity of diabetic gastroparesis underscores the importance of effective management strategies.

Diabetic gastroparesis usually persists once it has developed [14, 15]. Therefore, the most common GI adverse effects reported by patients using metformin tablets are diarrhea, nausea, and vomiting, with a prevalence of 2%–63%, which are experienced with metformin more frequently than those with any other oral antidiabetic medication [16, 17]. Although these GI side effects frequently go away with time and can be reduced by careful dose modification and taking metformin at mealtimes [18, 19], they may hinder compliance and lead to about 5% of patients stopping their prescriptions [17, 18]. Consequently, this study is aimed at estimating the prevalence of gastroparesis autonomic neuropathy in diabetic patients and determining the relationship between taking metformin and gastric symptoms in diabetic patients in Riyadh, Saudi Arabia.

## 2. Materials and Methods

### 2.1. Study Design and Sample Selection

A cross-sectional study was conducted using a Google Forms online survey with dual languages (Arabic and English). The decision to use Google Forms was made to facilitate data organization and security, allowing for efficient collection and analysis. The survey was distributed among randomly selected diabetic patients treated at a diabetic clinic in Prince Sultan Military Medical City, Riyadh, Saudi Arabia. The sample was selected from those who were visiting the diabetic clinic between March 7 and August 3, 2023, ensuring that each patient had an equal opportunity to participate. The survey was distributed among participants in the waiting area by trained medical students. All participants diagnosed with T1DM or T2DM and above the age of 18 were included in the study. When the survey was given, participants were asked to carefully read the purpose of the study before giving their consent. Patients were asked to provide consent before completing the survey, which was available in both Arabic and English to accommodate diverse participants. After the completion of the survey, the data was stored in a safe file that only authors had access to. All participants who refused to enroll in the study were excluded. The sample size was calculated through raosoft.com, which indicated 385 participants as a sufficient sample size to achieve a 5% margin of error and 95% confidence interval. While this sample size is adequate for estimating prevalence and detecting differences with a high level of confidence, it is important to acknowledge the potential for Type II errors of accepting a false null hypothesis, especially when detecting smaller effect sizes.

### 2.2. Questionnaire

The survey used the Gastroparesis Cardinal Symptom Index (GCSI); furthermore, it was revised by all authors to provide feedback on the survey sections and recommend any edits if needed. The questionnaire had three main sections. The first was about general background information such as age, gender, nationality, marital status, education, and employment. The second section was about diabetic medical history, which contained questions about the type of DM, disease duration, blood glucose control, and DM medication such as metformin. The third section was the GCSI. The GCSI has three subscales to measure the severity of symptoms related to gastroparesis: nausea/vomiting, postprandial symptoms/early satiety, and bloating. The nausea/vomiting subscale was measured by asking three about nausea, retching, and vomiting, while the postprandial fullness/early satiety subscale asked about stomach fullness, inability to finish a normal-sized meal, feeling excessively full after meals, and loss of appetite; also, the bloating subscale asked about bloating, and visibly larger stomach or belly after meals [19]. The GCSI consists of a total of nine questions, and each question is rated by the respondent on a scale of 0–5 depending on the severity of symptoms (0 = *no symptoms*; 5 = *severe symptoms*). Total GCSI scores were calculated by dividing the average of scores of the nine questions on 9. Scores of greater than 1.90 were selected as overt symptoms of gastroparesis. A cutoff of 1.90 was chosen according to data from previous literatures [19].

### 2.3. Data Entry and Statistical Analysis

Microsoft Excel and Statistics Package Social Science (SPSS) Version 25 (IBM Armonk, New York, NY, United States) were used for data entry and analysis. Graphical representation was generated using GraphPad Prism Version 9.4.1. To ensure a blind assessment of the outcome, an independent statistician performed the statistical analysis task. Categorical data were summarized using frequencies and percentages, while continuous data were presented in means and standard deviations. The normality of the data was determined with the Shapiro–Wilk test, which is appropriate for smaller sample sizes. If the data were normally distributed, a Student's *t*-test was used to evaluate differences in GCSI overall scores between two groups (e.g., gender, nationality, type of diabetes, controlled blood glucose levels, and metformin use), and a one-way ANOVA was used to test differences across more than two groups (e.g., age, marital status, education, employment, duration of diabetes, and use of other diabetes medications). For data that did not meet the normality assumption, nonparametric tests were applied, specifically the Mann–Whitney *U* test for two-group comparisons and the Kruskal–Wallis test for comparisons involving more than two groups. A *p* value of less than 0.05 was considered significant. Finally, multiple regression was used to predict the GCSI overall score based on various demographic characteristics. This analysis was chosen to account for the potential confounding effects of multiple variables simultaneously, allowing us to assess the independent contribution of each factor to the variation in GCSI scores.

### 2.4. Ethics Statement

The Institutional Review Board (IRB) committee at Prince Sultan Military Medical City, Scientific Research Center, has reviewed and approved this research with project number E-2113, dated July 13, 2023. The IRB-approved study was titled, “The Prevalence of Gastroparesis Among Diabetic Patients in Riyadh, Saudi Arabia.”

## 3. Results

The study cohort comprised 385 participants, with a relatively balanced gender distribution (52.2% male; 47.8% female). The vast majority of participants (97.7%) were of Saudi Arabian nationality (Table 1). Age distribution analysis revealed a predominance of individuals aged 60 years and above (34.0%), followed by those in the 18–29 year age bracket (23.4%), 50–59 years (21.8%), 40–49 years (12.2%), and 30–39 years (8.6%). With respect to diabetes classification, 214 participants (55.6%) were diagnosed with T2DM, while 171 (44.4%) had T1DM. Marital status distribution indicated that the majority of participants (69.0%) were married, with smaller proportions being single (23.6%), widowed (4.2%), or divorced (2.6%). Educational attainment varied among participants, with 38.2% (*n* = 147) having less than a high school degree and only 1.0% (*n* = 4) holding a doctoral degree. Employment status analysis revealed that a plurality of participants (36.4%) were retired, followed by students (16.9%), full-time employees working 40 or more hours per week (15.6%), housewives (13.8%), and part-time employees working 1–39 h per week (6.2%). Regarding disease duration, the majority of participants (59.55%) reported having diabetes for more than 10 years. The remaining participants were distributed as follows: less than 3 years (13.0%), 7–10 years (10.4%), 5–7 years (9.1%), and 3–5 years (8.1%). Notably, a substantial proportion of participants (56.6%) reported suboptimal glycemic control. Pharmacological management analysis revealed that approximately half of the participants (50.9%) were prescribed metformin. Other diabetes medications reported included insulin (41.6%), combination therapy of insulin and other agents (5.7%), sulfonylureas such as Diamicron (1.8%), Glucophage (0.5%), and sodium–glucose cotransporter-2 (SGLT2) inhibitors like Jardiance (0.3%). These demographic and clinical characteristics provide a comprehensive profile of the study population, enabling a nuanced interpretation of the subsequent analyses related to gastroparesis symptoms and their correlates. In Table 1, the demographic and clinical characteristics of study participants (*n* = 385) are presented.

Table 2 summarizes the frequency of gastroparesis symptoms among study participants. Among the GI symptoms, the item “stomach fullness” is the most frequent, with a prevalence of 53.2%. The least frequent is “vomiting,” with a prevalence of 17.9%. Furthermore, 41% of the participants suffered from nausea. About 21% of respondents complain of retching and 52.4% of feeling excessively full after meals. Also, 44.7% of participants answered that they were not able to finish a normal-sized meal, 43.4% suffered from loss of appetite, 47% from bloating, and 50.4%from stomach or belly visibly larger. Moreover, moderate levels of severity for “nausea,” “stomach fullness,” “feeling excessively full after meals,” “not able to finish a normal sized meal,” “bloating,” and “stomach or belly visibly larger” symptoms were more frequent than other levels of severity. Whereas, in “retching” and “loss of appetite” items, the highest frequent severity level is mild level. The overall median GCSI score for the sample population is 0.9 (0.2–1.9).

**Figure 1:**
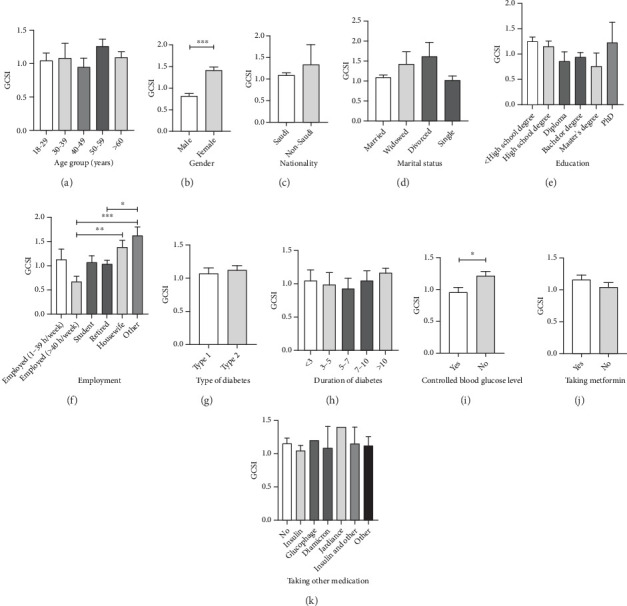
Differences in GCSI overall score based on study demographic groups of (a) age, (b) gender, (c) nationality, (d) marital status, (e) education, (f) employment, (g) type of diabetes, (h) duration of diabetes, (i) controlled blood glucose level, (j) taking metformin, and (k) taking other medication for diabetes mellitus. Data were expressed in bars showing means and standard error of measurements (SEMs). ⁣^∗^Significant difference from the control group (⁣^∗^*p* < 0.05, ⁣^∗∗^*p* < 0.01, and ⁣^∗∗∗^*p* < 0.001).

Differences in GCSI overall score based on study demographic groups are presented in Figure 1. The study showed that diabetic (AA8) gastroparesis which is measured by the GCSI score was similar among age groups, while female patients scored significantly higher rate with a mean of 1.411 (SD; 1.064) compared to male patients which gave a mean of 0.8179 (0.8688) (*p* value < 0.001). The groups choosing housewives with a mean of 1.385 (1.023) and other employment types, which include businessman, government employee, and unemployed, with a mean of 1.624 (1.127) were associated with significantly higher GCSI scores compared to other employment groups which include employed from 1–39 h per week and employed more than 40 h per week (*p* value < 0.05). Controlled blood glucose levels with a mean of 0.9575 (0.9532) were associated with significantly lower GCSI scores compared to noncontrolled blood glucose levels with a mean of 1.212 (1.041) (*p* value < 0.05). There were no significant differences in GCSI scores between the remaining demographic groups. Interestingly, there was no significant difference in the GCSI scores between T1DM with a mean of 1.073 (1.081) and T2DM with a mean of 1.125 (0.9527). Similarly, patients prescribed metformin with a mean of 1.162 (0.9913) exhibited similar GCSI scores compared to patients not receiving metformin with a mean of 1.039 (1.029) (*p* = 0.10).

**Figure 2:**
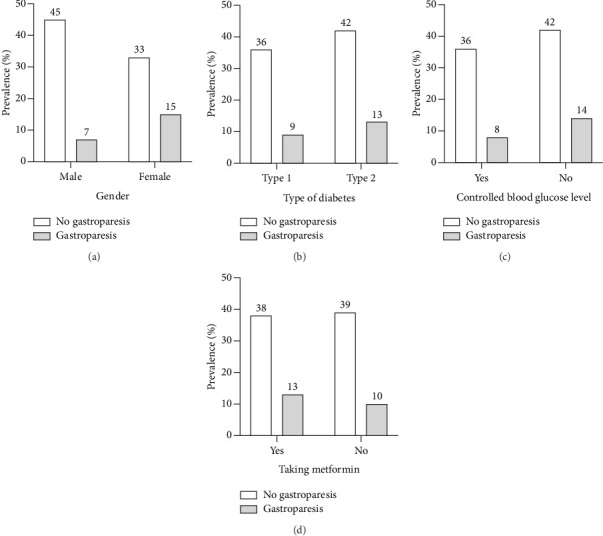
The prevalence of gastroparesis based on (a) gender, (b) type of diabetes, (c) controlled blood glucose level, and (d) taking metformin.

Prevalence of gastroparesis among diabetic patients based on gender, type of diabetes, controlled blood glucose level, and taking metformin are presented in Figure 2. Females showed a higher gastroparesis prevalence of 15% compared to males with 7%. Furthermore, uncontrollable blood glucose levels displayed a higher gastroparesis prevalence of 14% compared to controlled blood glucose levels with a prevalence of 8%. T2DM exhibited a marginally higher gastroparesis prevalence of 13% compared to T1DM with 9%. Moreover, taking metformin showed an increase of gastroparesis prevalence of 13% compared to non-taking metformin with a prevalence of 10%.

The conducted regression analysis aimed at exploring the relationship between demographic characteristics in predicting GCSI overall score (Table 3). *R* square of 0.128 indicates that independent variables (demographic characteristics) explain 12.8% of the variability of the GCSI overall score. The significant *F*-test indicates that the regression model is a good fit for the data, implying that the independent variables contribute to explaining the variability in the outcome (GCSI score). Only two independent variables showed significant association with the outcome; gender and controlled blood glucose level. The *t*-test statistic for gender was 4.629 (*p* value < 0.001), indicating that gender has a statistically significant effect on the outcome. Specifically, being female appears to be associated with a substantial increase in the dependent variable with an average score of 4.747 compared to the male group. The analysis showed that the uncontrolled blood glucose level group had a *t*-test statistic of 2.238 (*p* = 0.02), suggesting a significant association with an increase in the dependent variable. Other independent variables, such as age, nationality, marital status, education, employment, type of diabetes, duration of diabetes, taking metformin, and taking other medication, did not exhibit significant association with the outcome.

## 4. Discussion

Diabetic gastroparesis is the common term for upper GI symptoms associated with DM. This condition is characterized by delayed GE and upper GI symptoms that suggest, but are not related to, gastric outlet obstruction [19]. Symptoms of gastroparesis can vary widely, including bloating, nausea, vomiting, early satiety, and upper abdominal pain [18]. Historical data indicate that up to 60% of patients with long-standing T1DM and GI symptoms prior to the advent of intensive insulin therapy exhibited diabetic gastroparesis [17]. In contrast, the prevalence of gastroparesis in patients with T2DM varies, with estimates ranging from 10% to 30% in specialized hospital settings [20, 21]. As a result, this study is aimed at assessing the prevalence of gastroparesis among patients with T1DM and T2DM aged 18 years and older, as well as the relationship between metformin use and gastroparesis in T2DM patients. The present study identified a significant gender difference in GE, with females demonstrating a higher likelihood of experiencing gastroparesis compared to males, a finding consistent with previous research [22, 23].

A previous study reported that the risk of presenting at least one symptom of gastroparesis is five times greater in females [18]. Although the precise mechanism linking sex hormones to delayed gastric motility remains unclear, recent studies have highlighted the role of oxytocin in GE [24]. A literature review estimated that up to 40% of individuals with T1DM may experience gastroparesis, while the condition affects approximately 10%–30% of those with T2DM in tertiary care centers [25]). Similarly, our study corroborated these findings to some extent: Approximately 13% of T2DM patients exhibited clinical signs of gastroparesis according to the Gastroparesis Cardinal Symptom Index (GCSI) questionnaire, whereas only 9% of T1DM patients showed similar manifestations. These discrepancies may arise from the higher prevalence of diabetic complications observed in tertiary medical facilities compared to community-based surveys. These discrepancies can be due to the fact that tertiary medical facilities see a higher number of individuals with diabetic complications than community surveys do [25].

Additionally, our study found a marginal increase in the prevalence of clinical gastroparesis symptoms with diabetes, aligning with previous findings. This suggests that factors such as inadequate glycemic control, neuropathy, or delayed GE may contribute to the pathogenesis of gastroparesis [18].

While our study did not delve deeply into the differential impact of glycemic control on gastric function between insulin-dependent and insulin-independent diabetes, we did explore the relationship between controlled blood glucose levels and gastric symptoms. It is well-established that poor glycemic control can contribute to delayed GE [26, 27]. Our findings indicated higher GCSI scores among participants with suboptimal blood glucose control. Chronic hyperglycemia impairs GE and exacerbates gastroparesis symptoms, creating a reciprocal relationship wherein delayed GE further impairs calorie absorption and insulin release [28]. Some studies have reported no significant correlation between HbA1c levels and GE times [29, 30]. However, other studies found that higher HbA1c levels were associated with faster GE rates [31]. HbA1c reflects glycemic control over a three-month period, while gastroparesis may develop over a longer duration, potentially explaining these inconsistencies.

The results from our study indicate that there was no statistically significant difference in GCSI scores between patients on metformin and those not receiving the medication. Despite the lack of significance, patients prescribed metformin exhibited slightly higher GCSI scores compared to those not on metformin (mean GCSI 1.162 vs. 1.039, *p* = 0.10). This marginal difference aligns with previous studies, which have reported a notable association between metformin use and an increased risk of gastroparesis symptoms [1]. Metformin's potential role in gastroparesis development may be explained by its effect on the GI tract, primarily through its influence on glucagon-like peptide-1 (GLP-1) and dipeptidyl peptidase-4 (DPP-4). Metformin increases GLP-1 secretion, which is beneficial for glycemic control, but GLP-1 analogs are also known to cause GI side effects, including delayed GE, nausea, and vomiting. These side effects are characteristic of gastroparesis, suggesting that metformin's enhancement of GLP-1 could indirectly contribute to the exacerbation of gastroparesis symptoms in some patients [2].

Our study also revealed that metformin users had marginally higher GCSI scores compared to nonusers, which aligns with previous research linking metformin use to gastroparesis symptoms [32]. This association may be related to metformin's effects on increasing GLP-1 and inhibiting DPP-4, both of which are known to induce nausea and vomiting [33]. The lack of statistical significance in our findings could be due to the sample size or study design, which may have limited our ability to detect significant differences between the two groups. This is especially important given the known variability in GI responses to metformin, which could lead to smaller effect sizes that our study was underpowered to detect. Future studies should explore this relationship in larger cohorts and consider confounding factors such as the duration of metformin use, glycemic control, and the presence of other GI disorders. This would allow for a more comprehensive understanding of the relationship between metformin and gastroparesis in diabetic patients.

Our study is one of the first steps in finding the relationship between gastroparesis and DM. However, several limitations should be noted. First, the study used a relatively small sample size, which may limit the generalizability of the findings. While the sample size is sufficient for the primary analyses, it may not have been large enough to detect smaller effect sizes, increasing the risk of Type II errors. Second, as the study relied on a self-reported questionnaire, there is an inherent risk of recall bias, which may affect the accuracy of the data. Participants' self-assessment of their symptoms may vary based on their interpretation and tolerance levels, introducing variability. Moreover, although a random sampling technique was employed to select participants, reliance on patients attending the clinic during the study period may introduce some selection bias, as those present may not fully represent the broader diabetic population which may limit the generalizability of the results. Additionally, the diagnosis of gastroparesis in this study was not clinically validated by a physician, meaning the outcome was subjective and potentially prone to bias. Furthermore, the reliance on a Google Forms online survey may introduce selection bias, as it relies on patients who are comfortable using online tools and who attended the clinic during the study period. This may not fully represent the broader diabetic population. Future research could address these limitations by incorporating a more diverse and clinically validated sample and utilizing multiple methods of data collection to reduce bias.

In light of the limitations of our study, it is recommended that future research investigate a wide range of populations and settings by means of multicenter studies. This will allow for a more comprehensive expansion of perspectives and an improvement in the generalizability of the findings. Stronger evidence on the epidemiology, diagnosis, and symptomatology of gastroparesis could be obtained through the utilization of longitudinal designs or randomized controlled trials, which would contribute to improved health outcomes. The inherent bias of the cross-sectional design, which prevents the establishment of causal relationships between the use of metformin and the prevalence of gastroparesis, is one of the most significant limitations of our researchers' investigation. Furthermore, the use of self-reported survey data introduces the possibility of recall or reporting biases, which may have an effect on the accuracy of the findings. The fact that our research was conducted in Riyadh, which is a single center, also makes it difficult to generalize our findings to other populations.

Several questions still remain to be answered. In detail, studies frequently indicate that the severity and symptoms of gastroparesis can differ between these two groups, with T1DM being traditionally more closely associated with gastroparesis. Nevertheless, our findings, as well as recent research, suggest that the severity of symptoms as measured by GCSI may not be significantly different between T1DM and T2DM, which challenges previous assumptions. This may be attributable to improved glycemic control or advancements in diabetes management in both types, which could potentially reduce the severity of symptoms. The manuscript's future strategy is to explicitly reference and incorporate pertinent studies. The absence of a substantial difference in GCSI (Gastroparesis Cardinal Symptom Index) scores between T1DM and T2DM necessitates our investigation. This may be attributable to improved glycemic control or advancements in diabetes management in both types, which could potentially reduce the severity of symptoms.

## 5. Conclusion

In conclusion, this study is aimed at measuring the prevalence of gastroparesis among T1DM and T2DM patients and determining the relation between taking metformin and gastric symptoms in diabetic patients, which is the first step in finding the link between gastroparesis and DM. Our findings indicated higher GCSI scores in patients with uncontrolled blood glucose levels, female gender, metformin usage, and more than 10 years duration of diabetes. However, no variation between T1DM and T2DM in the GCSI score was observed. Our study underscores the need for future research on a larger population and obtaining a clinical diagnosis to eliminate interpretive bias.

## Figures and Tables

**Table 1:** Demographic characteristics of the study participant (*n* = 385).

**Variable**	**N** ** (%)**
Age	
18–29 years old	90 (23.4)
30–39 years old	33 (8.6)
40–49 years old	47 (12.2)
50–59 years old	84 (21.8)
≥ 60 years old	131 (34)
Gender	
Male	201 (52.2)
Female	184 (47.8)
Nationality	
Saudi	376 (97.7)
Non-Saudi	9 (2.3)
Marital status	
Married	268 (69.6)
Widowed	16 (4.2)
Divorced	10 (2.6)
Single (never married)	91 (23.6)
Education	
Less than high school degree	147 (38.2)
High school degree or equivalent	100 (26.0)
Diploma	29 (7.5)
Bachelor degree	87 (22.6)
Master's degree	18 (4.7)
PhD	4 (1.0)
Employment^a^	
Employed, working 1–39 h per week	24 (6.2)
Employed, working 40 or more hours per week	60 (15.6)
Student	65 (16.9)
Retired	140 (36.4)
Housewife	53 (13.8)
Other	41 (10.6)
Type of diabetes	
Type 1	171 (44.4)
Type 2	214 (55.6)
Duration of diabetes	
Less than 3 years	50 (13.0)
3–5 years	31 (8.1)
5–7 years	35 (9.1)
7–10 years	40 (10.4)
More than 10 years	229 (59.5)
Controlled blood glucose level	
Yes	167 (43.4)
No	218 (56.6)
Taking metformin	
Yes	196 (50.9)
No	189 (49.1)
Taking other medication for diabetes mellitus	
No	144 (37.4)
Insulin	160 (41.6)
Glucophage	2 (0.5)
Diamicron	7 (1.8)
Jardiance	1 (0.3)
Insulin and other	22 (5.7)
Other	49 (12.7)

^a^Two data are missing.

**Table 2:** Frequency of gastroparesis symptoms among study participants.

**Gastroparesis symptoms**	**Severity of the symptom, ** **N** ** (%)**					
	**None**	**Very mild**	**Mild**	**Moderate**	**Sever**	**Very severe**
Nausea	227 (59.0)	29 (7.5)	29 (10.1)	60 (15.6)	21 (5.5)	9 (2.3)
Retching	304 (79.0)	20 (5.2)	27 (7.0)	22 (5.7)	12 (3.1)	—
Vomiting	316 (82.1)	22 (5.7)	18 (4.7)	16 (4.2)	13 (3.4)	—
Stomach fullness	180 (46.8)	44 (11.4)	46 (11.9)	66 (17.1)	34 (8.8)	15 (3.9)
Feeling excessively full after meals	183 (47.5)	46 (11.9)	49 (12.7)	55 (14.3)	38 (9.9)	14 (3.6)
Not able to finish a normal-sized meal	213 (55.3)	31 (8.1)	34 (8.8)	56 (14.5)	37 (9.6)	14 (3.6)
Loss of appetite	218 (56.6)	43 (11.2)	48 (12.5)	41 (10.6)	22 (5.7)	13 (3.4)
Bloating	204 (53.0)	25 (6.5)	39 (10.1)	59 (15.3)	32 (8.3)	26 (6.8)
Stomach or belly visibly larger	191 (49.6)	36 (9.4)	42 (10.9)	55 (14.3)	36 (9.4)	25 (6.5)
GCSI overall score, median (IQR)	0.9 (0.2-1.9)					

Abbreviations: GCSI, Gastroparesis Cardinal Symptom Index; IQR, interquartile range.

**Table 3:** Multiple regression model for predicting GCSI overall score from demographic characteristics.

Regression model summary		
*R* square	0.128	
*F*-test (*p* value)	4.931 (< 0.001⁣^∗∗∗^)	

Independent variables coefficients		
Variable	Coefficient (B)	*t*-test (*p* value)
Age	−0.328	−0.665 (0.51)
Gender (reference; male)	4.747	4.629 (<0.001⁣^∗∗∗^)
Nationality (reference; Saudi)	4.088	1.345 (0.18)
Marital status	−0.243	−0.433 (0.67)
Education	−0.339	−0.919 (0.36)
Employment	0.561	1.336 (0.18)
Type of diabetes (reference; Type 1)	0.160	0.153 (0.88)
Duration of diabetes	0.414	1.277 (0.20)
Controlled blood glucose level (reference; yes)	2.020	2.238 (0.02⁣^∗^)
Taking metformin (reference; yes)	−1.646	−1.648 (0.10)
Taking other medication	0.086	0.386 (0.70)

⁣^∗^Significant difference from the control group; *p* < 0.05.

⁣^∗∗^*p* < 0.01.

⁣^∗∗∗^*p* < 0.001.

## Data Availability

The data that support the findings of this study are available on request from the corresponding author.
